# Giant Topological Hall Effect Across a Broad Temperature Window in Co‐Doped Mn_3_Sn Noncoplanar Antiferromagnets

**DOI:** 10.1002/advs.76226

**Published:** 2026-06-27

**Authors:** Mingqian Zhang, Xinyu Yao, Fangyi Qi, Yalei Huang, Jincang Zhang, Kun Zhao, Guixin Cao

**Affiliations:** ^1^ Materials Genome Institute State Key Laboratory of Advanced Refractories Shanghai University Shanghai China; ^2^ School of Physics Nanjing University of Science and Technology Nanjing China

**Keywords:** inverse triangular spin structure, noncollinear antiferromagnet, noncoplanar spin structure, topological Hall effect

## Abstract

The interplay between magnetism and topology in a geometrically frustrated noncollinear kagome lattice generate a real‐space Berry curvature and produce a topological Hall effect (THE). However, the absence of a room‐temperature THE severely hinders its application in spintronics. Here, a intrinsic THE is showed up to ∼1.64 µΩ·cm for *x* = 0.42 in Mn_3−_ *_x_* Co*_x_* Sn at 2 K with the field applied along *H* // *z*, ∼ 9 times of the reported maximum value in other noncoplanar antiferromagnetic materials. Such a THE across a broad wide temperature window up to 300 K, where it reaches a value of 0.45 µΩ·cm, ∼ 5 times of the reported maximum value in other noncoplanar antiferromagnetic materials. We attribute this giant THE to a robust intrinsic scalar spin chirality induced by magnetic Co doping. Our findings establish Co‐doped Mn_3_Sn as a unique platform for tailoring noncoplanar spin textures via doping to yield an unprecedented THE. This thereby enables the application of robust topological spin textures near room temperature, highlighting their significant potential in antiferromagnetic spintronics.

## Graphical abstract

Co doping in Mn_3_Sn transforms the spin texture magnetic state from a coplanar noncollinear magnetic state into a noncoplanar one, resulting in a large topological Hall effect (THE) over a wide temperature range (2–300 K). The THE arises from a noncoplanar spin texture, driven by lattice distortion and enhanced out‐of‐plane Dzyaloshinskii‐Moriyainteraction (DMI).

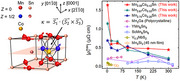

## 1 Introduction

Magnetic materials with topologically nontrivial spin textures have attracted widespread attention due to their unique transport phenomena and potential applications in spintronic devices [1, 2, 3, 4]. When electrons traverse nontrivial spin configurations arising from geometric frustration or antisymmetric Dzhaloshinskii‐Moriya interaction (DMI) [5, 6, 7, 8], an additional Hall resistivity is generated by the Berry curvature in real space, known as the topological Hall effect (THE). THE not only serves as an effective probe for detecting topologically nontrivial spin textures [9, 10], but also holds promise for application in spintronics and data storage technologies [11, 12, 13]. Two types of representative topological spin textures are currently recognized: skyrmion [14, 15, 16] induced by DMI interactions and noncoplanar spin structures resulting from the competition of various magnetic interactions. The latter has been reported in several materials, such as LaMn_2_Ge_2_ [17], YMn_6_Sn_6_ [18], MnBi_4_Te_7_ [19] and EuCuAs [20]. However, in most of these materials, THE only emerges at low temperatures, significantly limiting their utilities in practical applications in room‐temperature spintronic devices [17, 19, 20, 21, 22].

Chemical doping has proven to be an effective approach for tuning spin textures. By appropriate element substitution, coplanar noncollinear magnetic systems can be transformed to noncoplanar states that host THE, as demonstrated in Eu_1‐_ *_x_* Sm*_x_* TiO_3_ [23], Mn_3_Ga_0.8_Ge_0.2_ [24], (Fe*_x_* Co_1−_ *_x_*)_1/3_NbS_2_ [25], and Mn_3−_ *_x_* Fe*_x_* Sn [26]. For device applications, materials with robust perpendicular magnetic anisotropy and stable chiral spin textures at or above room temperature are highly desirable [27, 28]. This requires that the parent coplanar noncollinear magnetic materials possess a sufficiently high magnetic ordering temperature to enable THE to persist near room temperature. Among such candidates, Mn_3_Sn stands out with a high Néel temperature (*T*_N_ ≈ 420 K), making it a promising parent material for realizing room‐temperature THE and related spintronic functionalities [29, 30, 31]. Although stoichiometric Mn_3_Sn does not exhibit intrinsic THE, weak signals have been reported in polycrystals at low temperatures, attributed to field‐induced chiral spin textures and domain walls [32]. Substitution of Mn with magnetic dopants such as Co can induce out‐of‐plane magnetic moments, thereby forming noncoplanar spin configurations and giving rise to THE.

In this work, we systematically investigate the transport, magnetic and topological properties of Mn_3−_ *_x_* Co*_x_* Sn (*x* = 0, 0.26, 0.33, and 0.42) single crystals. For out‐of‐plane magnetic fields, Co‐substitution induces noncoplanar spin textures, leading to a pronounced THE across a broad temperature window (2−300 K). A giant THE of up to 1.64 µΩ·cm is observed in Mn_2.58_Co_0.42_Sn at 2 K. These findings highlight the critical role of electron doping in engineering noncoplanar spin configurations and provide valuable insights for the development of high‐performance spintronic devices capable of stable operation from cryogenic to room temperature.

## 2 Results and Discussion

**FIGURE 1:**
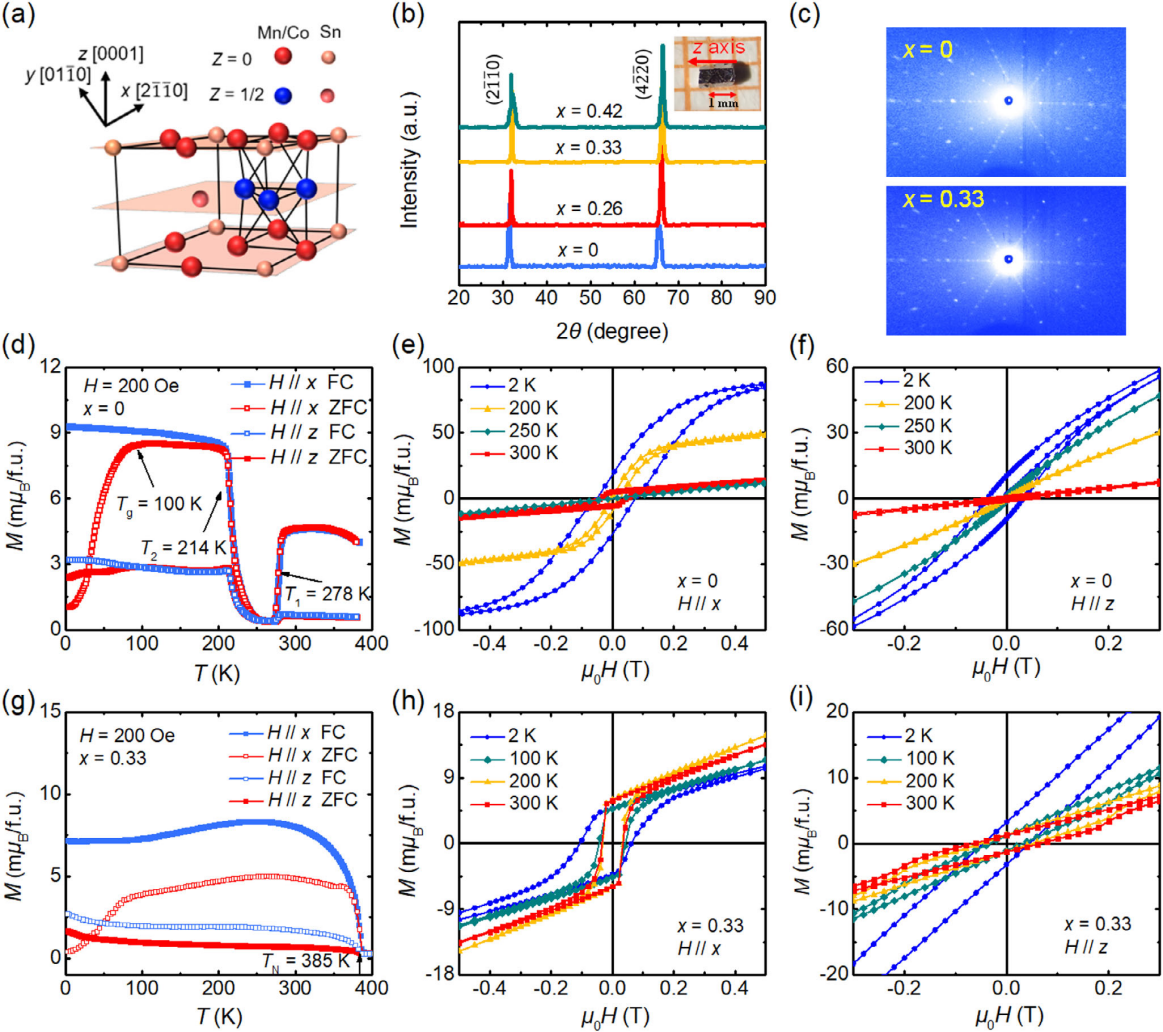
Structure and magnetic properties of Mn_3−_ *_x_* Co*_x_* Sn single crystals. (a) Crystal structure of Mn_3−_ *_x_* Co*_x_* Sn. (b) XRD pattern of the (2$\;\bar{1}\;\bar{1}\;$0) crystal plane for Mn_3−_ *_x_* Co*_x_* Sn. The inset shows a photo of Mn_2.67_Co_0.33_Sn single crystal. (c) Laue diffraction patterns of Mn_3−_ *_x_* Co*_x_* Sn single crystals in the [0 0 0 1] direction. (d) Temperature‐dependent magnetization *M*(*T*) measured under a magnetic field of 200 Oe along the *x*‐axis (*H* // *x*) and the *z*‐axis (*H* // *z*) in both FC and ZFC modes for *x* = 0. (e, f) *M*(*H*) measured at various temperatures with the magnetic field along the *x*‐axis (*H // x*) and the *z*‐axis (*H* // *z*) for *x* = 0. (g) *M*(*T*) measured under 200 Oe along the *x*‐axis (*H* // *x*) and the *z*‐axis (*H* // *z*) in both FC and ZFC modes for *x* = 0.33. (h, i) *M* (*H*) with the magnetic field oriented along the *x*‐axis (*H* // *x*) and the *z*‐axis (*H* // *z*) for *x* = 0.33, respectively.

Mn_3−_ *_x_* Co*_x_* Sn crystallizes in a hexagonal Ni_3_Sn‐type structure (space group *P*6_3_/*mmc*), as depicted in Figure 1a. The XRD patterns of Mn_3−_ *_x_* Co*_x_* Sn single crystals exhibit strong reflections from the $\left(2\bar{1}\bar{1}0\right)$ planes, with no detectable impurity peaks (Figure 1b), confirming the high crystalline quality. The Laue diffraction pattern along the [0001] direction (Figure 1c) further shows bright, sharp, and well‐defined diffraction spots, consistent with expectations for high‐quality single crystals. Combined with powder XRD results (Figure S1), these findings demonstrate that Co substitution systematically shifts diffraction peaks without altering the crystal structure.

**FIGURE 2:**
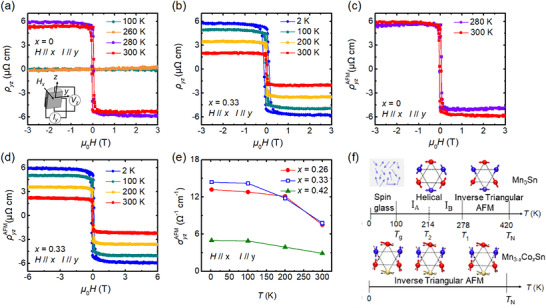
Berry‐curvature‐driven AHE for *H // x* at 2–300 K and magnetic phase diagram of Mn_3−_ *_x_* Co*_x_* Sn. (a, b) Field‐dependent Hall resistivity ρ_*yz*_ measured at various temperatures for (a) *x* = 0, (b) *x* = 0.33, respectively. The inset in panel (a) shows a schematic illustration of the measurement geometry for the *yz*‐plane Hall resistivity. (c, d) Field‐dependent AH resistivity $\rho_{yz}^{\mathrm{AFM}}$ extracted at various temperatures for (c) *x* = 0, (d) *x* = 0.33, respectively. (e) Temperature dependence of the AH conductivity. (f) Schematic magnetic phase diagram and corresponding spin structures of Mn_3−_ *_x_* Co*_x_* Sn.

Figure 1d,g presents the temperature‐dependent magnetization *M*(*T*) curves for *H* // *x* and *H* // *z*, respectively. For the parent Mn_3_Sn sample (*x* = 0), a sharp drop in magnetization occur at *T*_1_ = 278 K, corresponding to a first‐order magnetic transition from the inverse triangular spin structure to a helical configuration [33]. Around *T*_2_ = 214 K, the *M*(*T*) curves exhibits a ferromagnetic‐like transition. As the temperature decreases, the helical magnetic structure of Mn_3_Sn evolves through two successive spin states. The first helical state, I_B_, appears in the temperature range *T*_2_ < *T* < *T*_1_, with its magnetic easy axis along the *z*‐axis [0001]. Upon further cooling into the range *T*_g_ < *T* < *T*_2_, this spin state transitions into the second helical state, I_A_, whose magnetic easy axis lies along the *x*‐axis [11$\bar{2}$0] [33]. Below *T*_g_ = 100 K, a bifurcation between field‐cooled (FC) and zero‐field‐cooled (ZFC) curves indicates a spin‐glass‐like transition. The relevant magnetic phase diagram is shown in Figure 2f. In contrast, Co‐doping profoundly alters the magnetic properties: for *x* = 0.33, the *M*(*T*) curves show only a single antiferromagnetic transition at *T*_N_ = 385 K, with no signatures of the transitions at *T*_1_, *T*_2_, or *T*_g_ (see Figure 2f). The same trend is observed for other Co‐doped samples (Figure S2), confirming that Co‐doping suppress the first‐order magnetic transitions and stabilizes antiferromagnetic state.

The isothermal magnetization *M*(*H*) curves under *H* // *x* for *x* = 0 and 0.33 are shown in Figure 1e,h, respectively. For *x* = 0, hysteresis is evident above *T*_1_, arising from the weak ferromagnetism associated with the inverse triangular spin structure. Between *T*_1_ and *T*_2_, the hysteresis vanishes as the system enters a helical antiferromagnetic state, in agreement with *M*(*T*) results shown in Figure 1d. After the ferromagnetic‐like transition at *T*_2_, the *M*(*H*) curve develops a pronounced *S*‐shape. At *T* < *T*_g_, the *S*‐shaped hysteresis of the *M*(*H*) curve becomes more pronounced, reflecting enhanced ferromagnetic components tand competing interactions between ferromagnetic and antiferromagnetic exchange. This competition leads to partial spin freezing and the emergence of a spin‐glass‐like state. In contrast, for *x* = 0.33, *M*(*H*) exhibits the hysteresis behavior across the broad temperature range from 2 to 300 K, demonstrating that Co‐doping suppress the first‐order transition at *T*_1_ and stabilizes the inverse triangular spin structure.

The isothermal magnetization *M*(*H*) curves under *H* // *z* for *x* = 0 and 0.33 are shown in Figure 1f,i, respectively. The parent sample displays *M*(*H*) behavior similar with that for *H* // *x* (Figure 1f). However, in the Co‐doped samples, the *M*(*H*) curves show slight hysteresis near zero field (Figures 1i and S3), suggesting the emergence of weak out‐of‐plane ferromagnetic components. This phenomenon and the slight increase in out‐of‐plane magnetization indicate a gradual tilting of the magnetic moments away from the basal plane toward the *z*‐axis upon Co substitution.

To further elucidate the effect of Co‐doping on the electronic and topological responses, Hall measurements were performed along *H* // *x* on Mn_3−_ *_x_* Co*_x_* Sn single crystals. For the parent compound *x* = 0, the *ρ*(*H*) curves exhibit anomalous Hall effect (AHE) only above first‐order magnetic transition (*T*_1_ = 278 K) as shown in Figure 2a, where the inverse triangular structure transforms into a helical configuration [29, 34]. The inset of Figure 2a presents a schematic illustration of the measurement geometry for the *yz*‐plane Hall resistivity. In contrast, the *ρ_yz_* (*H*) curves of Co‐doped samples retain clear AHE signatures over a broad temperature form 2 to 300 K (Figures 2b and S4). Despite their weak net magnetization, the magnitude of AHE in Mn_3−_ *_x_* Co*_x_* Sn is comparable to that of ferromagnets [35], indicating that, the wide‐temperature‐range AHE in Co‐doped samples originates predominantly from Berry curvature effects in momentum space as in pristine Mn_3_Sn [36]. The Hall resistivity can be expressed as $\rho_{yz}=\rho_{yz}^{O}+\rho_{yz}^{A}+\rho_{yz}^{\mathrm{AFM}}=R_{0}\;\mu_{0}H+4\pi R_{s}M+\rho_{yz}^{\mathrm{AFM}}$ [29], where $\rho_{yz}^{O}$ and $\rho_{yz}^{A}$ are the ordinary and anomalous Hall (AH) resistivity, *R*_0_ and *R*_s_ are their corresponding coefficients, and $\rho_{yz}^{\mathrm{AFM}}$ is the AH resistivity driven by Berry curvature. The detailed procedure for extracting $\rho_{yz}^{\mathrm{AFM}}$ is provided in Figure S5. The saturation values of $\rho_{yz}^{\mathrm{AFM}}$ extracted at various temperatures (Figures 2c,d and S4) closely match the saturation values of *ρ*_*yz*_ (Figures 2a,b and S4), confirming that the AHE in Mn_3−_ *_x_* Co*_x_* Sn is Berry curvature dominated. To further prove this point, longitudinal magnetoresistivity (MR) measurements were performed with *H*∥*x* (Figures S4). Compared to the parent compound, the Co‐doped sample shows negative MR from 2–300 K, proving that Co doping stabilizes the inverse triangular spin structure and broadens the Weyl point window, leading to a wide‐temperature‐range AHE [37, 38]. The corresponding AH conductivity, 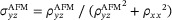 [39], is plotted in Figure 2e. For the Co‐doped samples, $\sigma_{yz}^{\mathrm{AFM}}$ is large at low temperatures due to reduced phonon scattering and enhanced carrier mobility [40], reaching a maximum of $\sigma_{yz}^{\mathrm{AFM}}$ ∼ 14.4 Ω^−1^·cm^−1^ at 2 K for *x* = 0.33. A notable feature is that the AH conductivity shows a nonlinear dependence on the doping concentration, which is attributed to magnetic doping altering the scattering intensity and leading to an amplification of longitudinal resistivity (Figure S5) [41]. Based on these findings, we summarize the magnetic phase evolution of Mn_3−_ *_x_* Co*_x_* Sn in Figure 2f.

**FIGURE 3:**
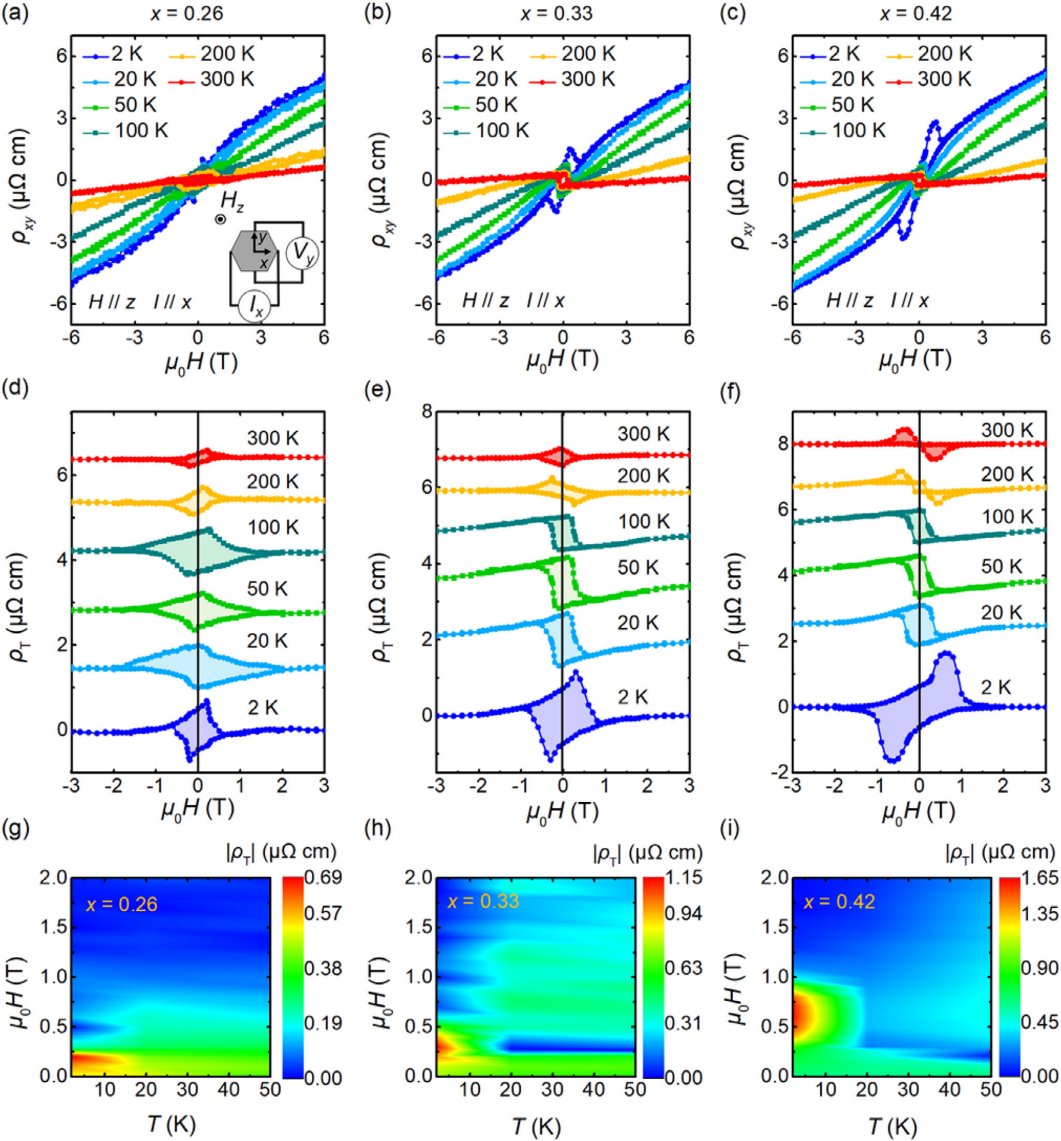
THE of Mn_3−_ *_x_* Co*_x_* Sn for *H // z* over 2−300 K. (a, b, c) Out‐of‐plane Hall resistivity (*H* // *z*, *I* // *x*) as a function of magnetic field at various temperatures for *x* = 0.26, 0.33 and *x* = 0.42, respectively. The inset in panel (a) shows a schematic illustration of the measurement geometry for the *xy*‐plane Hall resistivity. (d, e, f) Extracted topological Hall resistivity *ρ*_T_ as a function of magnetic field at different temperatures for *x* = 0.26, 0.33, and 0.42, respectively. The curves are vertically shifted for clarity. (g, h, i) Color maps of *ρ*_T_ as a function of temperature and magnetic field for *x* = 0.26, 0.33, and 0.42, respectively.

To further clarify the out‐of‐plane response, we investigated the Hall effect with *H* // *z*. The inset of Figure 3a presents a schematic illustration of the measurement geometry for the *xy*‐plane Hall resistivity. Unlike the linear field dependence observed in the parent sample (Figure S7), the *ρ*_*xy*_ of Co‐doped samples exhibits a pronounced hump‐like feature with a hysteresis loop over 2−300 K, a hallmark of the THE [42, 43, 44]. (Figure 3a–c) The Hall resistivity can be expressed as $\;\rho_{xy}=\rho_{xy}^{O}+\rho_{xy}^{A}+\rho_{T}=R_{0}\;\mu_{0}H+4\pi R_{s}M+\rho_{T}$ [29], where *ρ*_T _denotes the THE resistivity. By subtracting the ordinary and anomalous Hall components (Figure S7), the extracted THE resistivity (*ρ*_T _) is shown inFigure 3d–f. A maximum ρ_T_ value is observed at 2 K, and persists up to 300 K although its magnitude decreases with increasing temperature.

Since THE in noncoplanar magnets is proportional to the scalar spin chirality $\kappa =\vec{S_{1}}\cdot \left(\vec{S_{2}}\times \vec{S_{3}}\right)$ [45, 46], its magnitude strongly depends on the spin canting angle *θ* [31]. The rapid increase of *ρ*_T_ with increasing Co content in Mn_3−_ *_x_* Co*_x_* Sn at 2 K suggests that the electron doping enhances the canting angle of the spins. However, the non‐monotonic variation of *ρ*_T_ with temperature and Co‐doping highlights the complex interplay of spin chirality, electronic structure, and thermal effect. Notably, for *x* = 0.33 and 0.42, ρ_T_ undergoes a sign reversal around 200 K: positive‐to‐negative under positive magnetic fields and negative‐to‐positive under negative fields (Figure 3e,f). To visualize the variation of the THE, Figure 3g–i presents phase diagrams of *ρ*_T_ as a function of external magnetic field and temperature. The maximum ρ_T_ values reach 0.69, 1.15 and 1.64 µΩ·cm for *x* = 0.26, 0.33 and 0.42, respectively, underscoring the significant impact of Co‐doping in stabilizing noncoplanar spin structures and extending THE over a broad temperature window.

**FIGURE 4:**
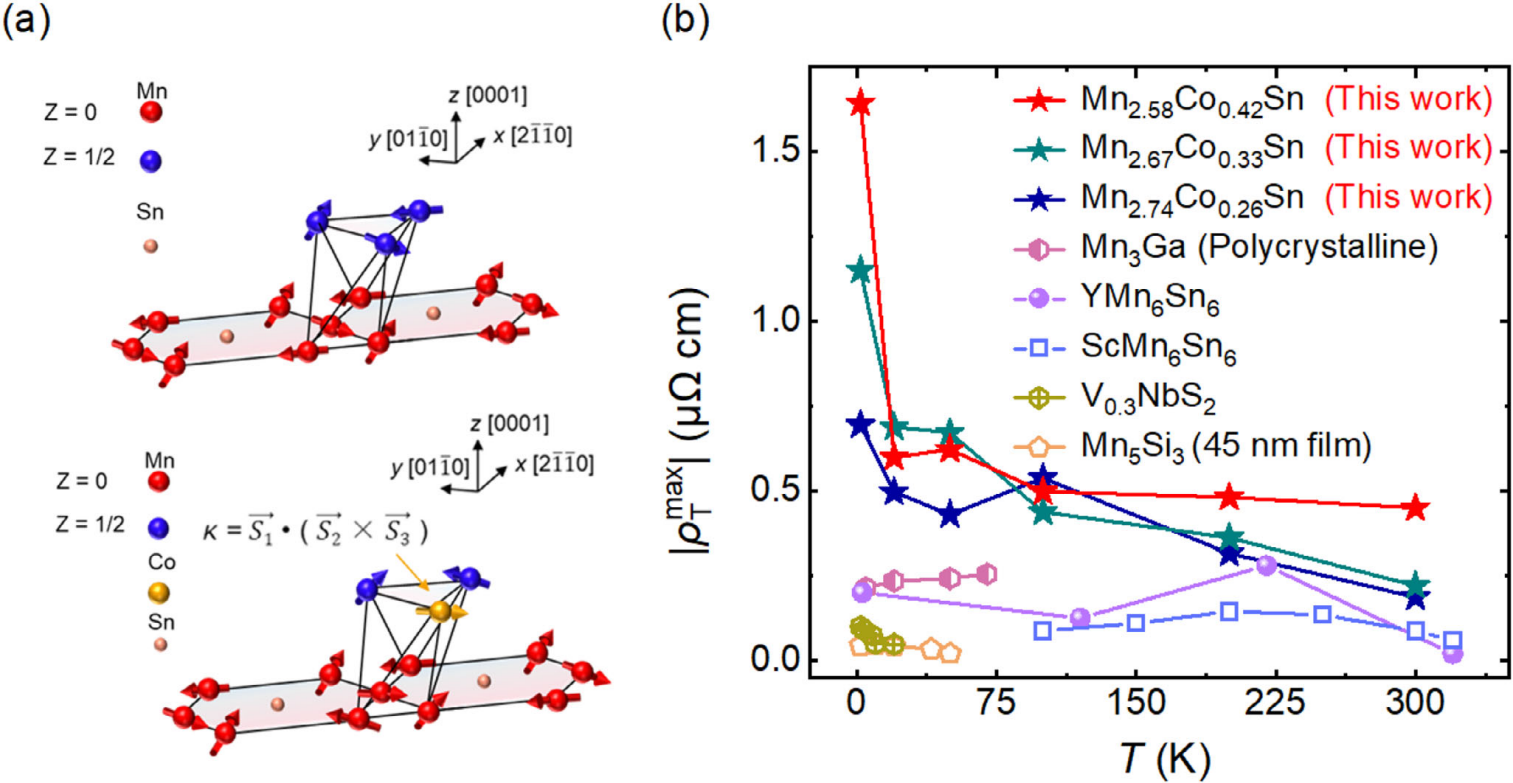
Spin magnetic structure of Co‐doped Mn_3_Sn and the comparison of topological Hall resistivity for Mn_3−_ *_x_* Co*_x_* Sn and different materials. (a) Schematic diagram of the spin magnetic structure for Mn_3_Sn. (b) Schematic diagram of the spin magnetic structure for Co‐doped Mn_3_Sn. (c) The comparison of topological Hall resistivity for Mn_3−_ *_x_* Co*_x_* Sn and different materials. Except for Mn_3−_ *_x_* Co*_x_* Sn, other data are taken from references [48−52]. Among them, the THE of YMn_6_Sn_6_ and ScMn_6_Sn_6_ is observed for *H* // *z*.

To connect these experimental results with the underlying mechanism, we present schematic models of the spin textures in Figure 4. For pristine Mn_3_Sn, the spins form a coplanar inverse triangular structure (Figure 4a). Upon Co‐doping, local lattice distortions associated with the Jahn‐Teller effect enhance the out‐of‐plane DMI [47]. This interaction drives a canting of the Mn moments out of the plane, producing finite out‐of‐plane magnetic moments and leading to the formation of noncoplanar spin configurations with non‐zero scalar spin chirality (Figure 4b). As a result, a sizable THE emerges. The magnitude of the observed THE in Mn_3−_ *_x_* Co*_x_* Sn is significantly larger than in other noncollinear antiferromagnets with relatively high *T*_N_, such as bulk YMn_6_Sn_6_ [48], ScMn_6_Sn_6_ [49], and Mn_3_Ga [50]. For instance, at 2 K, the topological Hall resistivity (ρ_T_) of Mn_2.58_Co_0.42_Sn reaches 1.64 µΩ·cm, which is about 9 times higher than that of YMn_6_Sn_6_ (0.18 µΩ·cm) [48]. At 300 K, the *ρ*_T_ value remains as high as 0.45 µΩ·cm for Mn_2.58_Co_0.42_Sn, which is about 5 times larger than the reported value of 0.086 µΩ·cm for ScMn_6_Sn_6_ [49]. While other chiral spin structure materials, such as V_0.3_NbS_2_, and Mn_5_Si_3_, also exhibit THE, their effects are typically restricted to narrower temperature ranges [51, 52]. In contrast, Mn_3‐_ *_x_* Co*_x_* Sn maintains robust THE across an unusually broad temperature window, extending to room temperature (Figure 4c). Furthermore, the observed THE in our study is realized with magnetic field applied along *H* // *z*, which is typically more beneficial for nanoscale spintronic devices, with advantages like thermal stability and low switching current density. The combination of large magnitude, wide temperature stability, and favorable field orientation makes Co‐doped Mn_3_Sn a promising candidate for spintronic devices.

Finally, motivated by the robustness of THE in Co‐doped Mn_3_Sn, we propose a conceptual design for a spintronic memory device that harnesses topological spin textures. As illustrated in Figure S8, the design integrates a spin‐valve write head, a voltage‐controlled magnetic anisotropy (VCMA) gate for pinning/depinning spin textures, and an magnetic tunnel junction (MTJ) ‐based read head for detection. While this schematic provides only a starting framework, the realization of such devices will require further optimization through simulation and experiment, particularly in improving spin current manipulation, enhancing VCMA efficiency, and developing more sensitive detection techniques. Nevertheless, the demonstration of room‐temperature THE in Mn_3‐_ *_x_* Co*_x_* Sn highlights its potential as a building block for future high‐performance spintronic memory technologies.

## 3 Conclusion

In summary, we have systematically investigated the effects of Co‐doping on the resistivity, magnetic, and topological properties of Mn_3_Sn. With the magnetic field applied along *H // z*, Mn_2.58_Co_0.42_Sn exhibits a remarkably large topological Hall resistivity, reaching 1.64 µΩ·cm at 2 K and remaining as high as 0.45 µΩ·cm at 300 K. This combination of exceptional magnitude and stability across a broad temperature range far surpasses other chiral spin‐structure materials. These results establish Co‐doped Mn_3_Sn as a unique platform for spintronics, offering both tunability and robustness of the THE, and highlight its strong potential for next‐generation device applications.

## 4 Experimental Section

### 4.1 Synthesis

Single crystals of Mn_3‐_ *_x_* Co*_x_* Sn (*x* = 0, 0.26, 0.33 and 0.42) were synthesized using the Sn‐flux method [53]. High‐purity powders of Mn (Alfa Aesar, 99.99%), Co (Alfa Aesar, 99.99%), and Sn (Alfa Aesar, 99.99%) were mixed in a molar ratio of 7:*γ*:3 (*γ =* 0, 0.8, 1.0, 1.3). The mixture was put in an alumina crucible and sealed in a vacuum quartz tube. The mixture was then heated to 1373K for 24 h, followed by slow cooling to 1123 K. After cooling, the quartz tube was decanted using a centrifuge to separate the Mn_3‐_ *_x_* Co*_x_* Sn crystals from excess Sn flux at this temperature. This process yielded several shiny hexagonal prism single crystals, with typical dimensions of 1.5 × 1 × 1 mm^3^, as shown in the inset of Figure 1b.

### 4.2 Characterization

The crystal structure and phase purity were characterized by X‐ray diffraction (XRD) using a Bruker D8 x‐ray diffractometer with Cu *K*𝛼 radiation (𝜆 = 0.15418 nm) at room temperature. The chemical compositions of the crystals were determined by energy‐dispersive X‐ray spectroscopy (EDX, HGSTFlexSEM‐1000), yielding compositions of Mn_3_Sn, Mn_2.74_Co_0.26_Sn, Mn_2.67_Co_0.33_Sn and Mn_2.58_Co_0.42_Sn, respectively.

### 4.3 Transport Measurement

Magnetization measurements were carried out using a superconducting quantum interference device magnetometer (MPMS, Quantum Design). The resistivity was measured using the standard four‐probe method by a physical property measurement system (PPMS‐14, Quantum Design).

## Author Contributions

G.C., K.Z., J.Z. conceptualized and designed the research, conducted data analysis, and revised the manuscript. M.Z., X.Y., F.Q, and Y.H. conduct experiments, data collection, data analysis, and interpretation. M.Z. and G.C. wrote the manuscript with the help of other authors. All authors participated in the discussion and contributed to scientific discussions and manuscript revisions.

## Conflicts of Interest

The authors declare no conflicts of interest.

## Supporting information




**Supporting File**: advs76226‐sup‐0001‐SuppMat.docx.

## Data Availability

The data that support the findings of this study are available from the corresponding author upon reasonable request.
