# Supplementary material for: Giant Topological Hall Effect Across a Broad Temperature Window in Co‐Doped Mn3Sn Noncoplanar Antiferromagnets
Source: Adv Sci (Weinh). 2026 Jun 27:e76226. Online ahead of print. doi: 10.1002/advs.76226 (PMC13336504; doi:10.1002/advs.76226)
Supplement: Supplementary file 1 — Supporting File: advs76226‐sup‐0001‐SuppMat.docx. [file ADVS-9999-e76226-s001.docx]

Supporting Information

**Giant topological Hall effect across a broad temperature window in Co-doped Mn_3_Sn noncoplanar antiferromagnets**

*Mingqian Zhang, Xinyu Yao, Fangyi Qi, Yalei Huang, Jincang Zhang*, Kun Zhao*, and Guixin Cao**

M. Zhang, X. Yao, F. Qi, Y. Huang, J. Zhang, G. Cao

Materials Genome Institute, State Key Laboratory of Advanced Refractories, Shanghai University, Shanghai 200444, China

E-mail: [guixincao@shu.edu.cn](mailto:guixincao@shu.edu.cn), jczhang@shu.edu.cn,

K. Zhao

School of Physics, Nanjing University of Science and Technology, Nanjing 210094, China

E-mail: [zhaokun@njust.edu.cn](mailto:zhaokun@njust.edu.cn)

**1. Powder X-ray diffraction pattern of Mn_3−_*_x_*Co*_x_*Sn**

Figure S1a shows the powder X-ray diffraction (XRD) pattern of Mn_3−_*_x_*Co*_x_*Sn single crystals. The substitution of Co atoms does not alter the overall crystal structure of Mn_3−_*_x_*Co*_x_*Sn. The lattice parameters calculated from the powder XRD results for the parent compound Mn_3_Sn, are *a* = 5.67 Å and *c* = 4.54 Å, which are consistent with previous reports.^[1]^ As the Co content increases, the (0002) diffraction peak exhibits a clear shift, as shown in the inset of Figure S1b, indicating changes in the lattice parameters with doping. Specifically, both *a* and *c* gradually decrease with increasing Co concentration as displayed in Figure S1b, which can be attributed to the smaller atomic radius of Co compared with Mn. The XRD patterns of Mn_3−_*_x_*Co*_x_*Sn single crystals exhibit strong reflections from the (0001) planes, with no detectable impurity peaks (Figure S1c), confirming the high crystalline quality. The elemental mapping of Energy-dispersive X-ray (EDX) spectra in Figure S1d confirms the presence of Mn, Sn, Co in the sample *x* = 0.26, and the distribution of all the elements is homogeneous, further confirming the successful doping of Co in Mn_3_Sn.


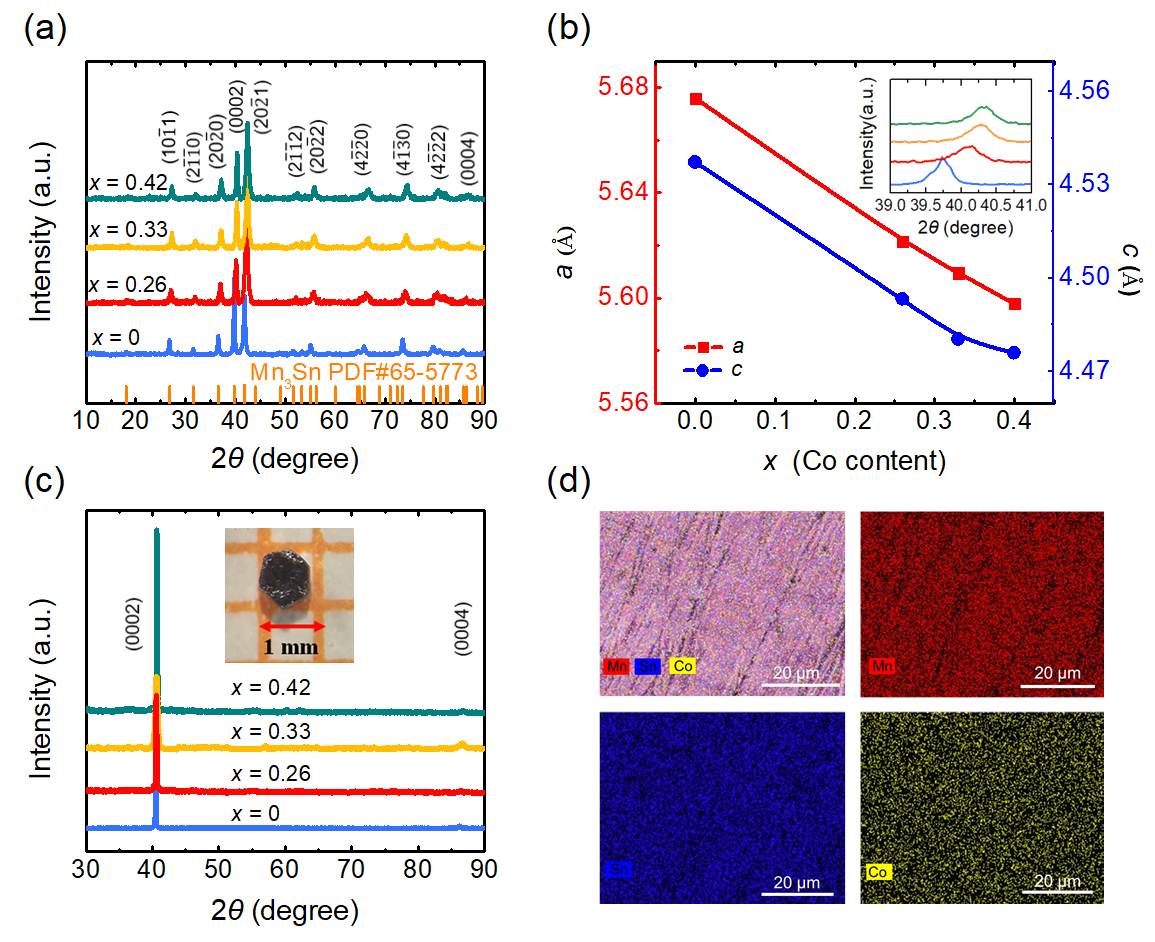


**Figure S1.** a) Powder XRD patterns of Mn_3−_*_x_*Co*_x_*Sn single crystals. b) Lattice parameters as a function of Co-doping concentration. The inset shows the (0002) diffraction peak for different Co-doped samples. c) XRD pattern of the (0001) crystal plane of Mn_3−_*_x_*Co*_x_*Sn single crystal. The inset shows a photo of Mn_2.67_Co_0.33_Sn single crystal. d) EDX surface mapping of the Mn_2.74_Co_0.26_Sn single crystals.

**2. *M*(*T*)** **for *x* = 0.26 and *x* = 0.42**

Figure S2 shows the temperature-dependent magnetization *M*(*T*) for *x* = 0.26 and *x* = 0.42. With Co-doping, both the first-order magnetic phase transition and the spin-glass-like transition disappear for *H // x* field orientations. As the magnetic field increases to 1 T (Figure S2c), the peak of the ZFC curve is suppressed, which is a characteristic of the antiferromagnetic phase transition. Co-substitution introduces ferromagnetic components and induces lattice distortion, thereby modifying the interactions among Mn atoms. As a result, the Néel temperature (*T*_N_) gradually decreases, from 389 K for *x* = 0.26 to 377 K for *x* = 0.42.


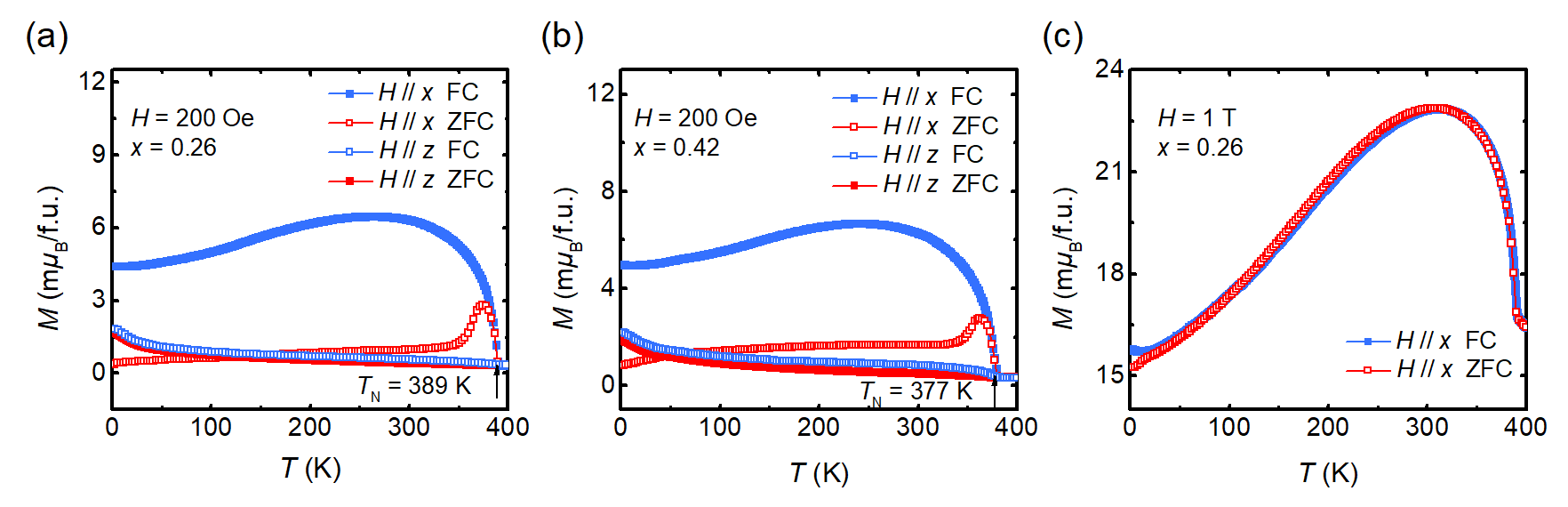


**Figure S2.** a, b) Temperature-dependent magnetization *M*(*T*) for *x* = 0.26 and *x* = 0.42 measured under *H* = 200 Oe in field-cooled (FC) and Zero-field-cooled (ZFC) modes with the magnetic field applied along the *x*-axis (*H*∥*x*) and the *z*-axis (*H*∥*z*). c) *M* (*T*) for *x* = 0.26 under *H* = 1 T, measured in FC and ZFC modes with the field applied along the *x*-axis (*H*∥*x*).

**3. *M*(*H*) for *x* = 0.26 and *x* = 0.42**

Figures S3a and S3b display the hysteresis associated with the inverse triangular spin structure induced by Co-doping in the temperature range of 2−300 K. Figures S3c and S3d present *M*(*H*) curves of Mn_3-_*_x_*Co*_x_*Sn for *H // z*. A weak hysteresis emerges near zero field, revealing the presence of a weak ferromagnetic component in the out-of-plane direction of the Co-doped samples.


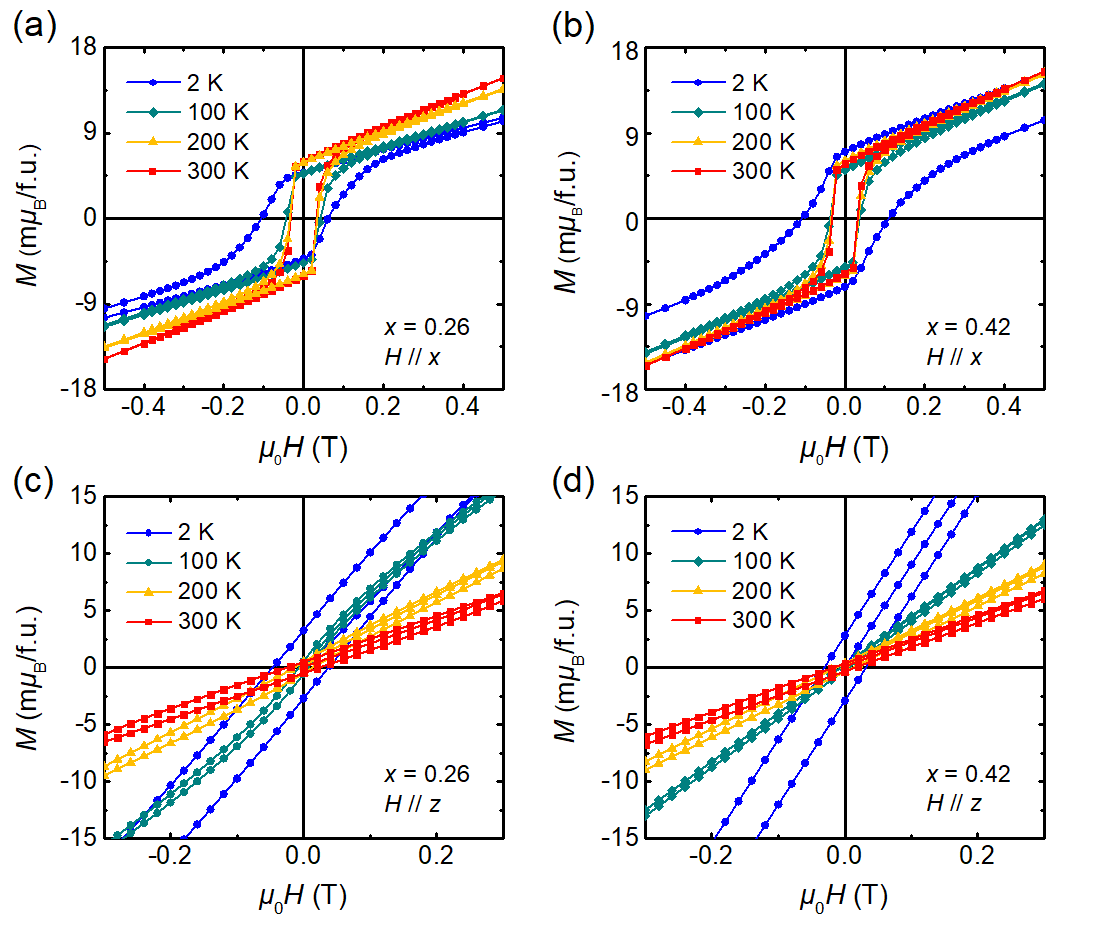


**Figure S3.** a, b) Magnetization isotherms *M*(*H*) of Mn_3−_*_x_*Co*_x_*Sn measured at different temperatures with the magnetic field applied along the *x*-axis (*H // x*) for *x* = 0.26 and *x* = 0.42. c, d) *M*(*H*) measured at different temperatures with the magnetic field applied along the *z*-axis (*H // z*) for *x* = 0.26 and *x* = 0.42.

**4.** **Hall resistivity and MR for Mn_3−_*_x_*Co*_x_*Sn**

Field-dependent Hall resistivity (*ρ_yz_*) for *x* = 0.26 and *x* = 0.42 measured at different temperatures for *H* // *x* axis are shown in Figures S4a and S4b. Both doped samples exhibit a clear anomalous Hall effect (AHE) over the range 2–300 K. The disappearance of the first-order magnetic phase transition due to Co-doping stabilizes the inverse triangular structure across this broad temperature window (2–300 K). The saturation values of $\rho_{yz}^{\mathrm{AFM}}$ for *x* = 0.26 and *x* = 0.42 at various temperatures (Figures S4c and S4d) are nearly identical to the corresponding saturation values of $\rho_{yz}$ shown in Figures S4a and S4b. Figure S4e shows that for the undoped sample (*x* = 0) at *T* = 300 K, the longitudinal magnetoresistance (MR) is negative. As the temperature decreases to 260 K, the negative MR switches to positive, indicating that the Weyl point vanishes once the *x* = 0 sample undergoes a first-order magnetic phase transition. In contrast, the *x* = 0.26 sample (Figure S4f) maintains negative longitudinal MR from 2 K to 300 K, suggesting that the AHE observed over this broad temperature range originates from Berry curvature associated with the Weyl point.


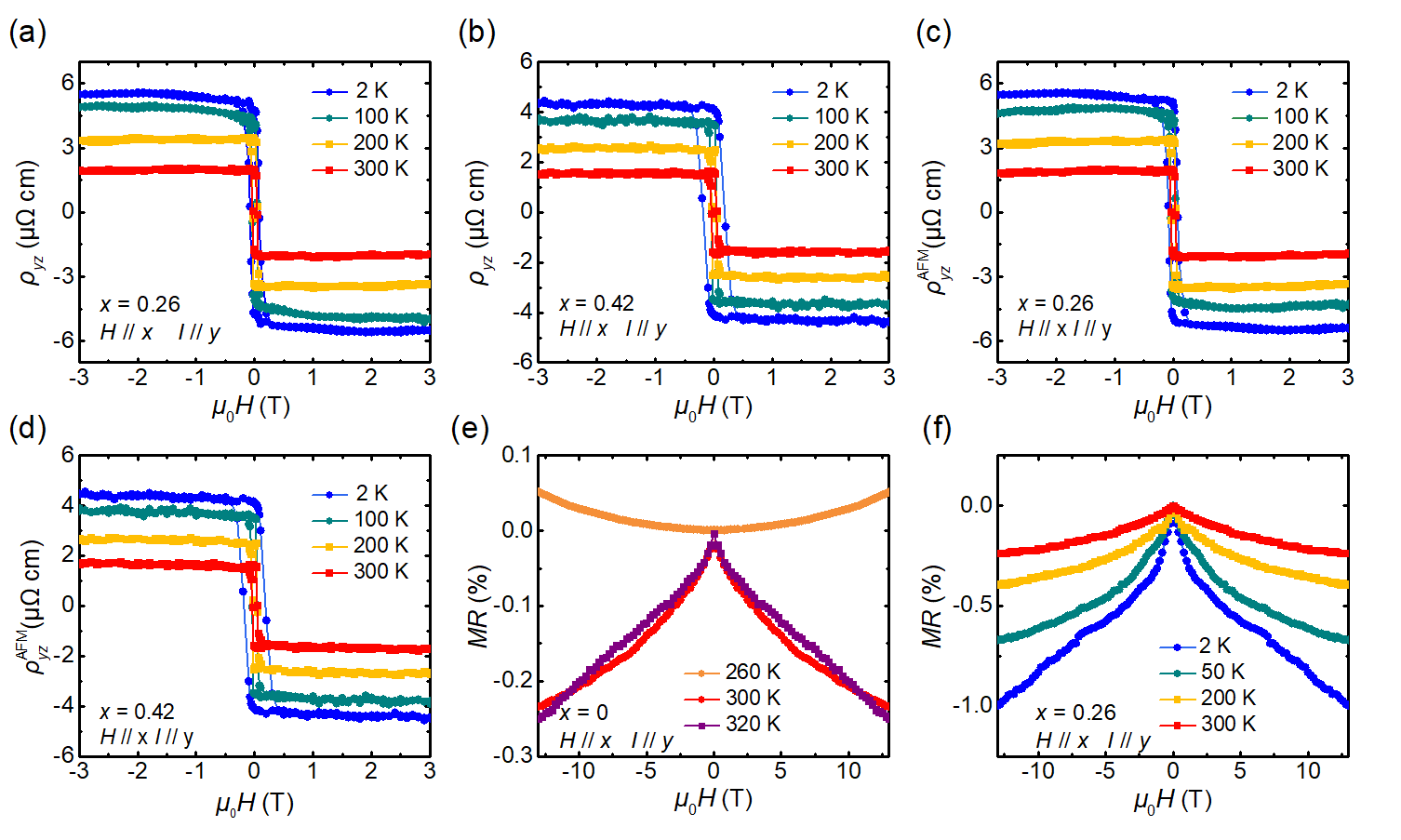


**Figure S4.** Field-dependent Hall resistivity of Mn_3−_*_x_*Co*_x_*Sn measured at different temperatures for a) *x* = 0.26 and b) *x* = 0.42 under *H*∥*x*. Field-dependent anomalous Hall resistivity $\rho_{yz}^{\mathrm{AFM}}$ measured at various temperatures for c) *x* = 0.26 and d) *x* = 0.42, respectively. Field-dependent MR measured at different temperatures for e) *x* = 0 and f) *x* = 0.26 with *H*∥*x*.

The Hall resistivity can be expressed as $\rho_{yz}= \rho_{yz}^{O}+\rho_{yz}^{A}+\rho_{yz}^{\mathrm{AFM}}=R_{0}\mu_{0}H+4\pi R_{s}M+\rho_{yz}^{\mathrm{AFM}}$. Taking the *x* = 0.42 sample as a representative example, the Hall resistivity of Mn_3-_*_x_*Co*_x_*Sn exhibits clear saturation in the high-field region. By performing a linear fit to the high-field region of the Hall resistivity as a function of the magnetic field, the slope directly yields *R*_0_ (Figure S5a). Similarly, by fitting the linear region of the Hall resistivity as a function of magnetization, the slope yields *R*_s_ (Figure S5b). After obtaining these two coefficients, $\rho_{yz}^{O}$and $\rho_{yz}^{A}$are subtracted from the total Hall resistivity to obtain the $\rho_{yz}^{\mathrm{AFM}}$ versus magnetic field curve (Figure S5c).


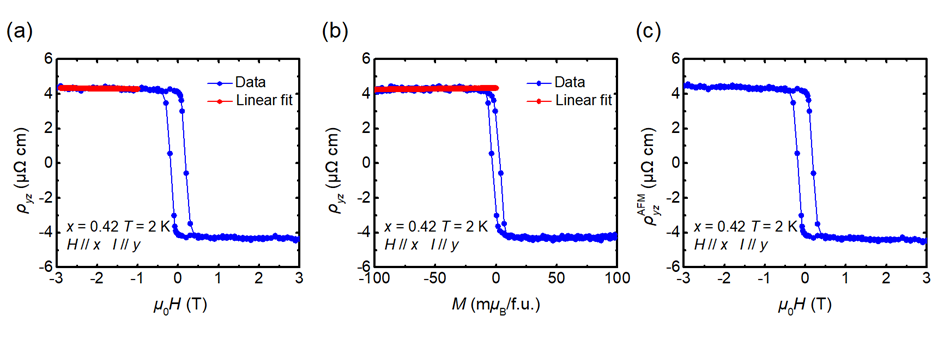


**Figure S5** a) The dependence of the Hall resistivity on the magnetic field at 2 K with the magnetic field applied along the *x*-axis (*H* // *x*) for *x* = 0.42. The red line represents the linear fit. b) The dependence of the Hall resistivity on the magnetization for *x* = 0.42 under *H*∥*x* at 2 K. The red line represents the linear fit. c) Field-dependent anomalous Hall resistivity $\rho_{yz}^{\mathrm{AFM}}$ measured at 2 K for *x* = 0.42.

**5. Longitudinal resistivity for Mn_3−_*_x_*Co*_x_*Sn**

Figure S6 depicts the zero-field longitudinal resistivity (*ρ_xx_*) of single crystals with *x* = 0.26, 0.33, and 0.42 measure in the temperature range 2–300 K. The Co-doped samples display a decrease in *ρ_xx_* with increasing temperature, which is a characteristic of semiconductor-like behavior. Similar resistivity trends have been reported in NiMnSn alloy^[2]^ and SrFeMoO_6_.^[3]^ This may arise from enhanced screening of the scattering potential, which occurs when the degree of disorder approaches a critical threshold.


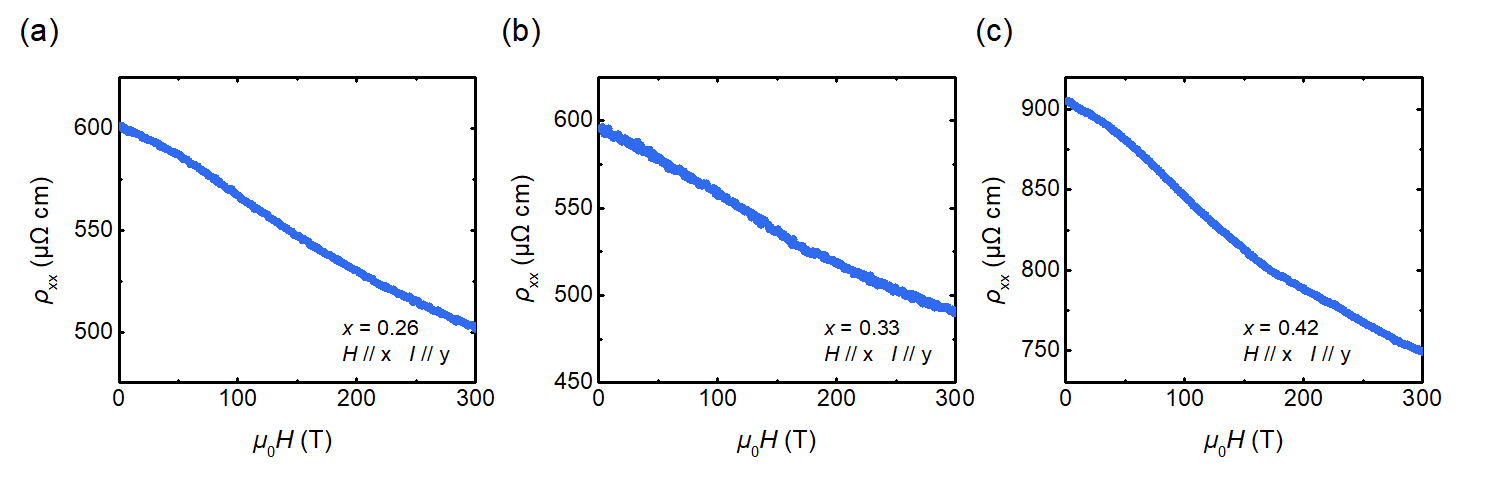


**Figure S6.** Temperature dependence of *ρ_xx_* for a) *x* = 0.26, b) *x* = 0.33, and c) *x* = 0.42 with the magnetic field applied with *x*.

**6. Ordinary Hall coefficient**

For *x* = 0, no AHE is observed, as evidenced by the linear relationship between Hall resistance and magnetic field when *H* // *z* (Figure S7a), consistent with previous reports.^[4]^ This behavior is attributed to the fact that all Mn magnetic moments in Mn_3_Sn lie within the *xy*-plane. The Hall resistivity can be expressed as$\rho_{xy}=\rho_{xy}^{O}+\rho_{xy}^{A}+\rho_{T}=R_{0}\mu_{0}H+4\pi R_{s}M+\rho_{T}.$ As the magnetic field increases, the non-coplanar spin structure is disrupted, leading to the disappearance of finite scalar spin chirality. Consequently, the THE vanishes, and thus *ρ*_T_ becomes zero in the high-field region. Under this condition, the expression can be rewritten as: $\frac{\rho_{xy}}{\mu_{0}H}$ = $R_{0} + R_{s}\frac{4\pi M}{\mu_{0}H}$. By performing a linear fit to the $\frac{\rho_{xy}}{\mu_{0}H}$-$\frac{M}{\mu_{0}H}$ curve in the high-field region (Figure S7b), the slope corresponds to 4π*R*_s_, yielding *R*_s_, and the intercept gives *R*_0_. The measured total Hall resistivity *ρ*_H_ includes contributions from the ordinary Hall effect, AHE and topological Hall effect (THE) (Figure S7c). The temperatures dependent of *R*_0_ and *R*_s_ are shown in Figure S7d.


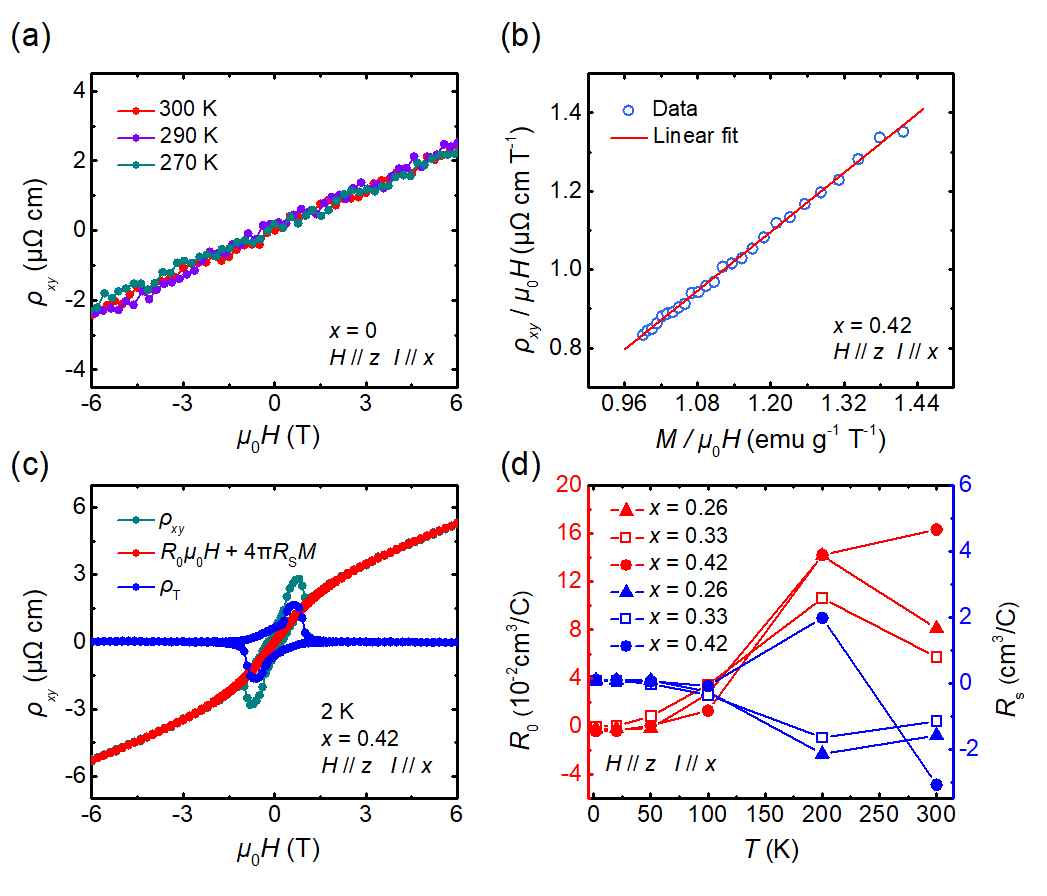


**Figure S7.** a) Hall resistivity curves for *x* = 0 at different temperatures with the magnetic field applied along the *z*-axis (*H*∥*z*). b) The sample-dependent $\rho_{xy}/\mu_{0}H$ vs $M/\mu_{0}H$ curve for the *x* = 0.42 sample, where the red solid line represents the linear fitting result. c) Contributions of each component to the Hall resistivity as a function of magnetic field for *x* = 0.42 at 2 K with *H*∥*z*. d) Temperature dependence of *R*_0_ and *R*_s_ for *x* = 0.26, *x* = 0.33 and *x* = 0.42.

**7. Schematic of memory device**

Figure S8 presents a schematic diagram of the proposed memory device. The topological spin texture is generated by injecting a local spin-polarized current (*I*_nucl_) through the spin-valve write head. By tuning the perpendicular spin current (*I*_driv_), the spin textures can be manipulated to arrange and move along nano-tracks. During this process, the textures can be pinned or depinned at the voltage-controlled magnetic anisotropy (VCMA) gate. Finally, a detection current (*I*_det_) is applied to probe and analyze the spin textures using magnetic microscopy or other highly sensitive magnetic measurement techniques. It should be noted that this schematic provides only a conceptual framework and practical implementation will require detailed simulation, experimental validation, and iterative optimization to overcome technical challenges.


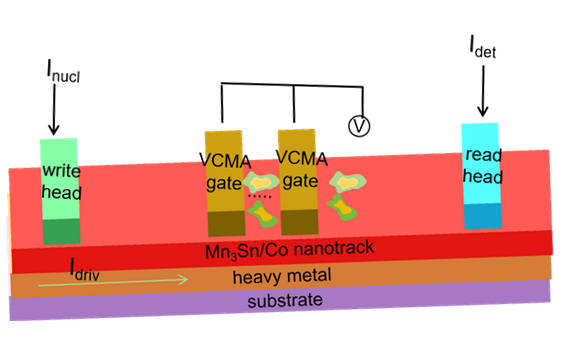


**Figure S8.** Schematic of the memory device, consisting of a spin-valve write head, nanotrack, magnetic tunnel junction read head, and a VCMA gate.

[1] H. Narita, M. Ikhlas, M. Kimata, A. A. Nugroho, S. Nakatsuji, Y. Otani, *Appl. Phys. Lett.* **2017**, 111, 202404.

[2] T. Fichtner, G. Kreiner, S. Chadov, G. H. Fecher, W. Schnelle, A. Hoser, C. Felser, *Intermetallics* **2015**, 57, 101.

[3] K.-I. Kobayashi, T. Kimura, H. Sawada, K. Terakura, Y. Tokura, *Nature* **1998**, 395, 677.

[4] S. Nakatsuji, N. Kiyohara, T. Higo, *Nature* **2015**, 527, 212.
